# Heart rate variability with circadian rhythm removed achieved high accuracy for stress assessment across all times throughout the day

**DOI:** 10.3389/fphys.2025.1535331

**Published:** 2025-04-14

**Authors:** Yafei Shen, Zihan Fang, Tao Zhang, Feng Yu, Ying Xu, Ling Yang

**Affiliations:** ^1^ School of Mathematical Sciences, Soochow University, Suzhou, Jiangsu, China; ^2^ Center for Systems Biology, Soochow University, Suzhou, Jiangsu, China; ^3^ Jiangsu Province Engineering Research Center of Molecular Target Therapy and Companion Diagnostics in Oncology, Suzhou Vocational Health College, Suzhou, Jiangsu, China; ^4^ Jiangsu Key Laboratory of Neuropsychiatric Diseases and Cambridge-Su Genomic Resource Center, Suzhou Medical College of Soochow University, Suzhou, Jiangsu, China

**Keywords:** stress assessment, heart rate variability, circadian rhythm, feature extraction, XGBoost

## Abstract

**Background:**

Assessing real-time stress in individuals to prevent the accumulation of stress is necessary due to the adverse effects of excessive psychological stress on health. Since both stress and circadian rhythms affect the excitability of the nervous system, the influence of circadian rhythms needs to be considered during stress assessment. Most studies train classifiers using physiological data collected during fixed short time periods, overlooking the assessment of stress levels at other times.

**Methods:**

In this work, we propose a method for training a classifier capable of identifying stress and resting states throughout the day, based on 10 short-term heart rate variability (HRV) feature data obtained from morning, noon, and evening. To characterize the circadian rhythms of HRV features, heartbeat interval data were collected and analyzed from 50 volunteers over three consecutive days. The circadian rhythm trends in the HRV features were then removed using the Smoothness Priors Approach (SPA), and XGBoost models were trained to assess stress.

**Results:**

The results show that all HRV features exhibit 12-h and 24-h circadian rhythms, and the circadian rhythm differences across different days for individuals are relatively small. Furthermore, training classifiers on detrended data can improve the overall accuracy of stress assessment across all time periods. Specifically, when combining data from different time periods as the training dataset, the accuracy of the classifier trained on detrended data increases by 13.67%.

**Discussion:**

These findings indicate that using HRV features with circadian rhythm trends removed is an effective method for assessing stress at all times throughout the day.

## 1 Introduction

Psychological stress refers to a mental state in which an individual feels pressured, tense, or uncomfortable in response to external stressors. This state can result from various challenges, threats, or changes that require the individual to adapt and respond (Folkman, 2013; Selye, 1958). While moderate psychological stress can be beneficial by enhancing motivation, increasing alertness, and improving focus, excessive psychological stress may have detrimental effects. It can contribute to cardiovascular diseases such as hypertension and heart disease, disrupt normal immune function, and cause hormonal imbalances (Cohen et al., 2007; Al-Shargie et al., 2017). Therefore, continuous monitoring of stress and timely intervention are key to managing both physical and mental health.

In recent years, the widespread use of smart wearable devices has facilitated the collection and analysis of physiological signals. An increasing number of researchers are focusing on leveraging these physiological signals to objectively detect psychological stress, enabling timely alerts regarding users’ stress levels (Marques et al., 2010). Currently, the primary physiological signals utilized for stress detection include electromyographic signals, electroencephalographic signals, electrocardiographic signals, and electrodermal activity signals (Healey and Picard, 2005; Singh et al., 2013; Peng et al., 2012; Al-Shargie et al., 2016). Among these, heart rate variability (HRV), derived from ECG signals, has gained increasing utilization in the assessment of psychological stress in recent years, owing to its ease of measurement.

Existing research indicates that stress disrupts the homeostasis of sympathetic and parasympathetic nervous system activity, thereby altering the oscillations of the cardiac cycle (Pereira et al., 2017; Petrowski et al., 2010; Camm et al., 1996a; Mukherjee et al., 2011; Luque-Casado et al., 2016; Beauchaine and Thayer, 2015). HRV, calculated from the intervals between two consecutive R-wave peaks (RR intervals) on an ECG, can effectively reflect these oscillations (Camm et al., 1996a). HRV features encompass a set of statistical metrics that provide insights into heart activity across time domain, frequency domain, and non-linear. During periods of psychological stress, significant alterations in HRV features are observed (Acerbi et al., 2016; Hynynen et al., 2011; Cinaz et al., 2013; Taelman et al., 2011; Tharion et al., 2009; Blechert et al., 2006; McDuff et al., 2016; Bernardi et al., 2000; Lucini et al., 2002; Schubert et al., 2009; Melillo et al., 2011). HRV analysis can be conducted over different time intervals, such as 24 h (referred to as long-term HRV analysis), 5 min (referred to as short-term HRV analysis), or even shorter intervals (Camm et al., 1996b). Research findings indicate that both long-term and short-term HRV features reliably reflect stress-related changes in real-life situations (Castaldo et al., 2015; Hernando et al., 2016; Delaney and Brodie, 2000; Salai et al., 2016; Munla et al., 2015). In the research process, the choice between long-term and short-term HRV largely depends on the research question and the type of stress being studied (Malik et al., 1996). In response to short-term stress, the autonomic nervous system (ANS) is influenced, leading to significant changes in HRV (Castaldo et al., 2015), which subsequently return to normal levels. Given its ability to capture rapid fluctuations in ANS activity, short-term HRV has been shown to be particularly suitable for studying short-term stress (Pereira et al., 2017; Cipresso et al., 2019).

Over the past few years, to better identify short-term stress and mitigate the health risks associated with stress accumulation, short-term HRV analysis (ranging from 5 to 60 s) has become increasingly utilized. The widespread adoption of wearable sensors in devices such as smartphones and smartwatches has further facilitated the convenience of short-term HRV analysis (Athavale and Krishnan, 2017; Pecchia et al., 2018). Castaldo et al. were the first to propose a rigorous methodology for assessing the effectiveness of ultra-short-term HRV features in detecting psychological stress. They demonstrated that HRV analysis can reliably and accurately detect psychological stress when transitioning from short-term (as a reference) to ultra-short-term intervals (Castaldo et al., 2019). Karthikeyan et al. extracted ultra-short-term (32-s) HRV features, including both time-domain and frequency-domain parameters. They used the Fast Fourier Transform (FFT) to select optimal features for model input, and, based on PNN and KNN algorithms, identified the best smoothing factor and K value, achieving high accuracy in classifying stress and normal states (Karthikeyan et al., 2013). Salahuddin et al. utilized the Stroop Color Word Test (SCWT) as a stress-inducing task and successfully collected data from 60 volunteers in both resting and stress states. Using statistical methods, they analyzed changes in various time-domain and frequency-domain features of ultra-short-term HRV under different conditions, providing a theoretical foundation for the effective monitoring of short-term stress (Salahuddin et al., 2007).

In addition to stress, the influence of circadian rhythms on sympathetic and parasympathetic nervous system activity has been increasingly reported in recent years (Niwa et al., 2011; Okada et al., 2013). Naturally, HRV and other physiological indicators have also been observed to follow circadian rhythms (Lee et al., 2023; Stangherlin et al., 2020). However, many existing studies either overlook the effects of these rhythms or mitigate them by selecting a fixed short time window for experimental assessments of stress based on HRV. For instance, Hemakom et al. developed a machine learning model to classify various stress levels using ECG and EEG data, with data collection limited to the hours between 10 a.m. and 12 p.m. to control for circadian influences (Hemakom et al., 2024). Similarly, da Estrela et al. studied low high-frequency HRV to determine whether they can predict stress-related sleep disturbances. To account for diurnal variations in HRV, all testing sessions were conducted in the morning (da Estrela et al., 2020). Despite these efforts, classifiers trained on physiological data collected during fixed short time periods often perform poorly when applied to stress assessment across other times of the day. Hayano et al. demonstrated that, due to the presence of both circadian and ultradian rhythms, applying short-term HRV analysis methods to long-term HRV data collected in free-living conditions can lead to inaccurate conclusions (Hayano and Yuda, 2021). Therefore, it is essential to mitigate the influence of circadian rhythms when evaluating stress based on HRV.

The analysis of the above literature motivated us to explore whether HRV data, with circadian rhythm trends removed, could be effectively used to train a classifier capable of accurately assessing stress levels across all time periods. In this study, we employed detrended short-term HRV features to evaluate stress. To capture circadian rhythm trends, we collected RR interval data from 50 volunteers continuously over 3 days, during which stress was induced using the SCWT task (Karthikeyan et al., 2014; Al-Shargie et al., 2022; Stroop, 1992). We then applied a smoothing prior method to eliminate the circadian rhythm trend from the HRV feature data. Subsequently, we trained XGBoost classifiers using both raw and detrended HRV feature data collected during the SCWT test, confirming the effectiveness of the detrended HRV features for assessing stress throughout the day. The influence of age and participants’ familiarity with the experiment on the final classification accuracy was also investigated. We proposed this approach primarily to enhance the accuracy of stress recognition, making it more applicable in real-life scenarios.

## 2 Materials and methods

Briefly, the specific process we established for developing a classifier to assess stress is as follows:

1. Data collection: RR interval data from 50 volunteers were collected continuously over 3 days using smartwatches, with stress induced through the SCWT task at 8:30 a.m., 2:00 p.m., and 10:30 p.m. each day.
2. Data preprocessing: Confidence ellipses were used to remove outliers from the collected RR interval data.
3. HRV feature extraction: HRV feature data for each individual was calculated throughout the day based on the denoised RR interval data. Subsequently, circadian rhythm trends in the HRV feature data were obtained and removed.
4. XGBoost classifier training: The detrended HRV feature data from the SCWT test sessions were integrated into a training set, and an XGBoost model was trained as a classifier to assess stress.

**FIGURE 1:**
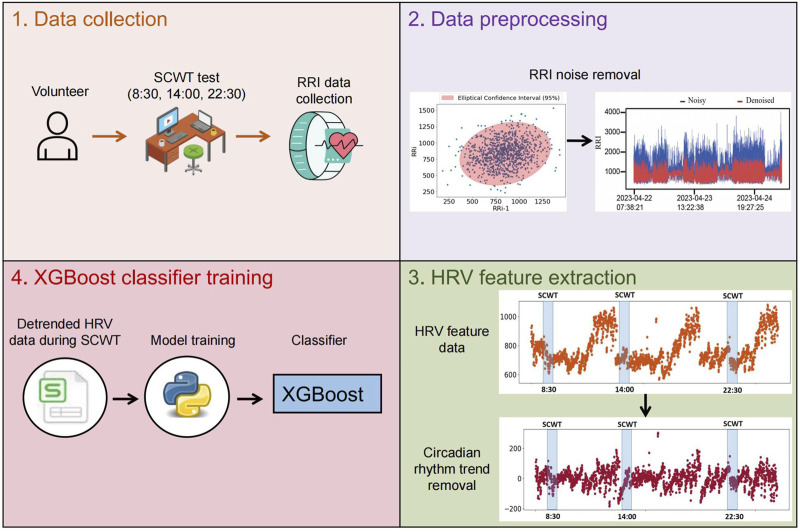
The workflow for obtaining the classifier that identify stress across all times throughout the day. The XGBoost model is used as a classifier and trained on detrended HRV feature data from the SCWT experiment.

Figure 1 provides an overview of the experimental workflow. Below are the specific details of each experimental step.

### 2.1 Participants

**FIGURE 2:**
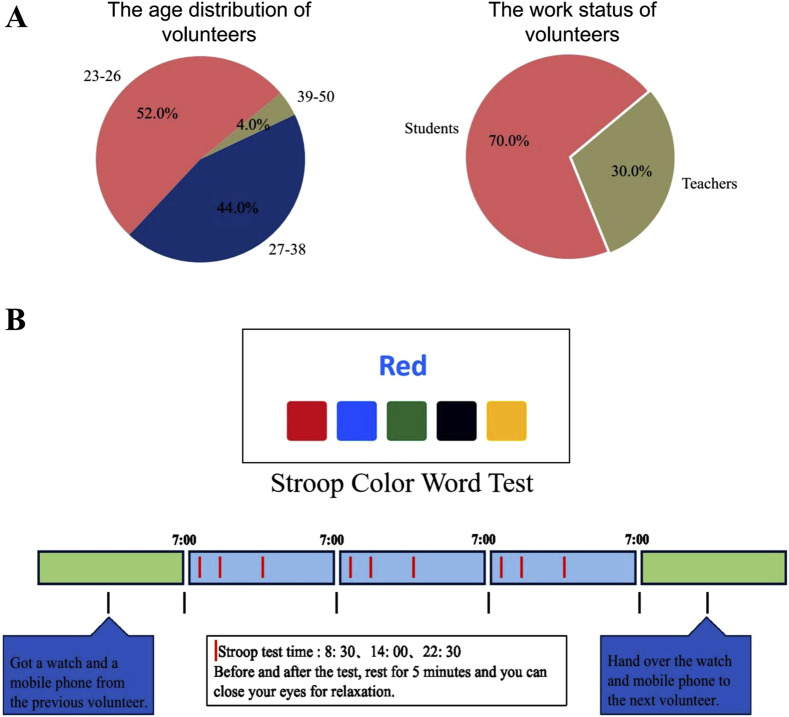
Details of data collection. **(A)** The age distribution and work status of volunteers participating in the SCWT experiment. **(B)** The process of the SCWT task. The RR interval data for each volunteer was collected over three consecutive days (marked by blue blocks). A Stroop Color Word Test was conducted at 8:30 a.m., 2:00 p.m., and 10:30 p.m. each day to induce stress (indicated by red vertical lines within the blue blocks).

During the data collection phase, fifty healthy volunteers (22 males and 28 females, aged 23–50) were recruited based on pre-screening questionnaires confirming good overall health, absence of psychological and cardiovascular diseases, and no color blindness or color weakness. These participants, comprising students and teachers, contributed heartbeat interval (RR interval) data for subsequent HRV analysis (as shown in Figure 2A). To comprehensively capture circadian rhythm information, RR interval data were collected continuously over a period of 3 days from each participant using a smartwatch. If a participant interrupted the data collection process on any given day, they were asked to reschedule and complete the data collection on a different day. The experimental procedure posed minimal impact on participants and did not interfere with their daily activities. Ethical approval for the study was obtained from the Ethics Committee of Soochow University (Approval No. SUDA20230828H01).

### 2.2 Experiment setup and task sequence

To collect data under stress conditions, stress was induced using the SCWT task (Karthikeyan et al., 2014; Al-Shargie et al., 2022; Stroop, 1992). To examine the effect of circadian rhythms on HRV, each participant completed the SCWT task 9 times over the 3 days, specifically at 8:30 a.m., 2:00 p.m., and 10:30 p.m. daily. Each SCWT session consisted of three phases (as shown in Figure 2B). Step 1 (resting phase): Participants were instructed to relax for 5 min prior to the task, either by closing their eyes or listening to soft music. Step 2 (stress phase): Participants then performed the SCWT on a computer, where a word describing a color was displayed with the font color differing from the word’s meaning. Below the word were five colored blocks. The participants were required to click the block that matched the color described by the word. To ensure pressure conditions during the test, participants are required to complete at least 140 questions within 2 min, with no more than two errors. If these criteria are not met, an immediate retest at the same time point is administered. The total response time must not exceed 5 min; otherwise, the test session is considered invalid. Step 3 (resting phase): Following the test, participants were given time to relax by sitting quietly to alleviate any residual stress.

In the SCWT test, the incongruent relationship between the word meaning and its font color could easily mislead participants, making it challenging to select the correct color block. This required participants to maintain high levels of concentration to complete the word-color matching tasks quickly and accurately, effectively inducing psychological stress. Furthermore, resting phases were incorporated before and after the SCWT test to minimize the influence of confounding factors, such as physical exertion, on the experimental outcomes.

### 2.3 Data acquisition

To successfully obtain the collected data, RR interval data from all volunteers were continuously recorded over a 3-day period using a smartwatch (Huawei Watch GT 2, green light, reflection pattern). The sensors used in this experimental equipment could measure ECG to capture RR interval data, with precision down to the millisecond. Besides, collecting ECG data using a smartwatch is very convenient and has minimal impact on participants’ daily lives. Around midnight each day, the collected data from the previous day was uploaded to the database via Bluetooth. Finally, we downloaded all the experimental data from the database for subsequent analysis.

### 2.4 Pre-processing

After the data collection was completed, we accounted for the potential impact of daily activities on the collection of RR interval data—such as intense wrist movements during activities like running, which can cause the smartwatch to produce unstable measurements, leading to missed or extra heartbeat values and, in turn, excessively large or small RR intervals—we converted the collected one-dimensional RR interval data into two-dimensional data. Specifically, the first dimension represents the previous RR interval value, $RR_{i}$ , and the second dimension represents the subsequent adjacent RR interval value, $RR_{i+1}$ , where i=1,2,…,N . The final two-dimensional dataset obtained was $\left\{\left(RR_{1},RR_{2}\right),\left(RR_{2},RR_{3}\right),\ldots ,\left(RR_{N-1},RR_{N}\right)\right\}$ , where N is the total number of RR intervals. Since the distribution of these two-dimensional data points was approximately elliptical, we applied confidence ellipses to remove noise from the data.

Generally, the mathematical expression for an ellipse is:

$${\left(\frac{x-R_{1p}}{a}\right)}^{2}+{\left(\frac{y-R_{2p}}{b}\right)}^{2}=1,$$

where $\left(R_{1p},R_{2p}\right)$ is the center of the ellipse. The covariance matrix of the transformed two-dimensional RR interval data is:

$$C=\left[\begin{array}{cc} COV\left(R_{1},R_{1}\right) & COV\left(R_{1},R_{2}\right)\\ COV\left(R_{2},R_{1}\right) & COV\left(R_{2},R_{2}\right) \end{array}\right],$$

where $COV\left(R_{i},R_{j}\right)$ represents the covariance between the variables $R_{i}$ and $R_{j}$ . Then, the elliptical equation that measures the error of each data point can be derived.

$$\frac{{\left(R_{1}-R_{1p}\right)}^{2}}{\lambda_{1}}+\frac{{\left(R_{2}-R_{2p}\right)}^{2}}{\lambda_{2}}=s,$$ (1)

$\lambda_{1}$ and $\lambda_{2}$ are the largest and smallest eigenvalues of the covariance matrix C , respectively, and s is the scale of the ellipse. Additionally, based on the chi-square distribution, we can obtain P(s<5.991)=0.95 . Therefore, we set the value of s to be 5.991 to obtain a 95% confidence ellipse. According to Equation 1, the long axis of the confidence ellipse is length $2\sqrt{5.991\lambda_{1}}$ , and the short axis is length $2\sqrt{5.991\lambda_{2}}$ . If we denote the eigenvector corresponding to the largest eigenvalue $\lambda_{1}$ of the covariance matrix as $\nu_{1}$ , then the angle between the major axis of the ellipse and the positive direction of the x-axis is:

$$\alpha =\arctan\frac{\nu_{1}\left(y\right)}{\nu_{1}\left(x\right)}.$$

$\nu_{1}\left(y\right)$ and $\nu_{1}\left(x\right)$ respectively represent the magnitudes of the projections of $\nu_{1}$ onto the positive y-axis and the positive x-axis.

Finally, based on the center of the ellipse $\left(R_{1p},R_{2p}\right)$ , the eigenvalues $\lambda_{1}$ and $\lambda_{2}$ of the data covariance matrix, the scale of the ellipse s , and the angle α between the major axis and the x-axis, we can establish a confidence ellipse to filter out noise from the data.

**FIGURE 3:**
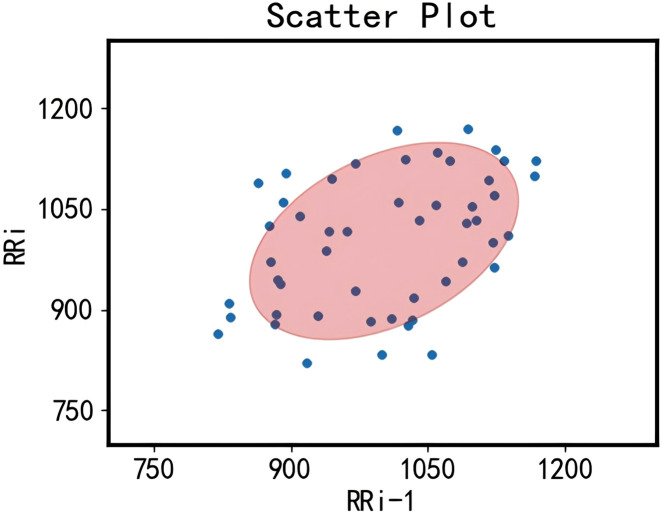
Diagram illustrating the removal of outliers in RR intervals using confidence ellipses. The blue points represent the distribution of the transformed two-dimensional RR interval data, where the values of each point’s two dimensions correspond to the lengths of two consecutive RR intervals. The red area indicates the 95% confidence ellipse interval.

We randomly selected 50 data points from the collected RR interval dataset to illustrate the results of noise removal using confidence ellipses. As shown in Figure 3, the two-dimensional RR interval data within the confidence ellipse indicate that the differences between adjacent RR intervals are minimal. In contrast, data points outside the confidence ellipse exhibit significant differences between adjacent RR intervals and are therefore considered noise to be removed.

**FIGURE 4:**
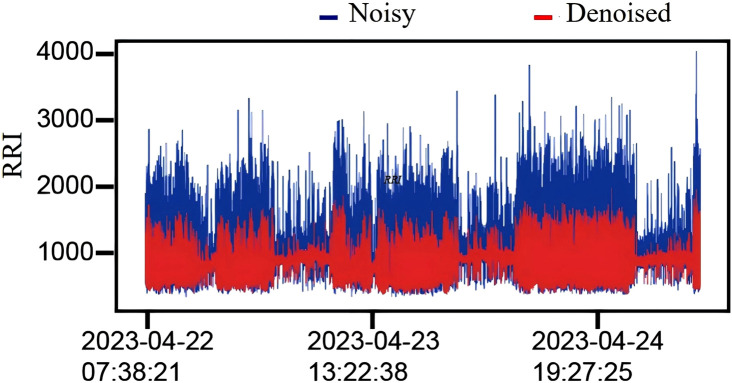
Effect of outlier removal. Comparison of 72-h RR interval data (RRI) before and after outlier removal for a volunteer. The blue part represents the data distribution before outlier removal, and the red part represents the data distribution after outlier removal.

**FIGURE 5:**
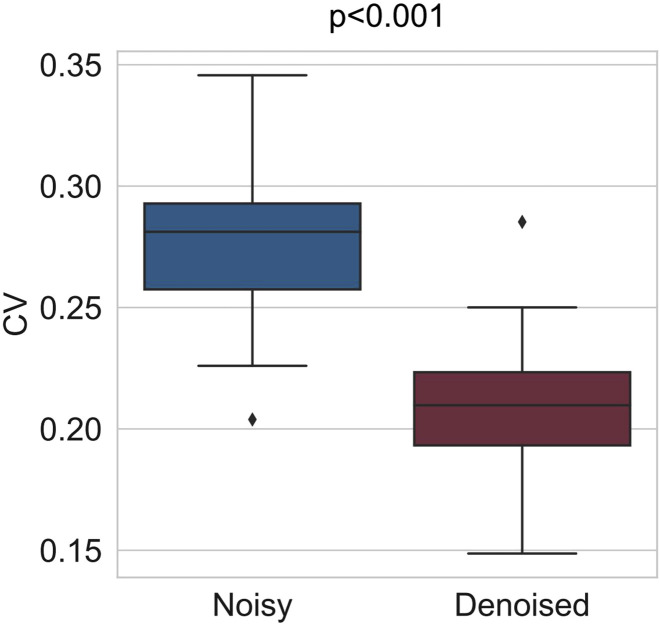
Comparison of CV coefficients before and after outlier removal for the RR interval data of all volunteers. After outlier removal, the average CV coefficient decreased from 0.28 to 0.2.

In this work, we employed a sliding window confidence ellipse method to remove noise from each volunteer’s RR interval data. The window length was set to 50 data points, with an overlap of 25 data points between consecutive windows. Specifically, the first 50 data points of each volunteer’s RR interval data were used to form the initial window. After applying the confidence ellipse to remove noise, the window was shifted back by 25 points to create the second window, where the confidence ellipse was established again for noise removal. This procedure was repeated until the window encompassed all data points. Figure 4 illustrates the results of the noise removal process applied to one volunteer’s RR interval data. The blue line represents the distribution of the original RR intervals, which exhibits significant fluctuations, while the red line indicates the distribution of the RR intervals following noise removal, showing a much smoother profile. Additionally, we quantified the dispersion of the RR interval data in the dataset by calculating the coefficient of variation (CV), using the following formula:

$$CV=\frac{\sigma }{\mu },$$

where σ is the standard deviation and μ is the mean of the RR interval data. Figure 5 displays the CV of the original RR interval data across all volunteers (average CV = 0.28) and the CV of the RR interval data following noise removal (average CV = 0.2). These results indicate that the sliding window confidence ellipse method effectively reduced abnormal fluctuations in the RR intervals.

### 2.5 Feature extraction

To evaluate HRV based on the collected RR interval data, we introduced five statistical metrics to extract the time-domain features of the RR interval data. The mean (meanNN) was used to reflect the average level of the RR intervals. The calculation formula is as follows:

$$meanNN=\bar{RR}=\overset{N}{\underset{i=1}{\sum }}\frac{RR_{i}}{N},$$

where N is the number of RR intervals within a 2-min period, $\bar{RR}$ is the average value of the RR intervals within a 2-min period and $RR_{i}$ represents the $i^{th}$ RR interval.

The average heart rate (HR) was used to reflect the average level of heart rate.

$$HR=\frac{\sum_{i=1}^{N_{1}}\frac{N_{1}}{RR_{i}}+\sum_{i=1}^{N_{2}}\frac{N_{2}}{RR_{i}}}{2},$$

$N_{1}$ and $N_{2}$ represent the number of RR intervals in the first and second minutes, respectively.

The overall standard deviation (SDNN) was introduced to reflect the overall variation in HRV:

$$SDNN=\sqrt{\frac{1}{N}\overset{N}{\underset{i=1}{\sum }}{\left(RR_{i}-\bar{RR}\right)}^{2}},$$

and the root mean square of successive differences (RMSSD) was used to estimate the fast component variations in HRV:

$$RMSSD=\sqrt{\frac{1}{N-1}\overset{N-1}{\underset{i=1}{\sum }}{\left(RR_{i+1}-RR_{i}\right)}^{2}}.$$

Additionally, we used the proportion of the number of successive RR interval differences greater than 50 milliseconds to the total number of RR intervals (PNN50) to reflect sudden changes in RR intervals. The calculation formula is as follows:

$$PNN50=\frac{NN50}{TotalNN},$$

where NN50 represents the number of successive RR interval differences greater than 50 milliseconds, and TotalNN represents the total number of RR intervals within a 2-min period.

To perform frequency domain analysis of HRV based on the RR interval data, we first estimated the power spectral density (PSD(f)) using an autoregressive (AR) model. Subsequently, we integrated the power spectrum over different frequency bands to extract three frequency domain features. The details of the power spectral density estimation are provided in Supplementary Material. Based on the frequency spectral density, we extracted the very low frequency power (vlf) .

$$vlf=\int_{0.0033}^{0.04}PSD\left(f\right)df,$$

low frequency power (lf)

$$lf=\int_{0.04}^{0.15}PSD\left(f\right)df,$$

and high frequency power (hf)

$$hf=\int_{0.15}^{0.5}PSD\left(f\right)df.$$

To further analyze HRV from a nonlinear perspective, we extracted two parameters based on the Poincare plot of the two-dimensional RR interval data (Brennan et al., 2001). The first parameter is the length of the minor axis (ST1) of the scatter plot distribution area within a specified time period, which is associated with vagal nerve activity. The second parameter is the length of the major axis (ST2) of the scatter plot distribution area within the same time period, which is indicative of changes in sympathetic nervous system activity. Their calculation formulas are as follows:

$$ST1=\sqrt{\frac{1}{N-1}\overset{N-1}{\underset{i=1}{\sum }}{\left(RR_{i}+RR_{i-1}-2\bar{RR}\right)}^{2}}.$$

$$ST2=\sqrt{\frac{1}{N-1}\overset{N-1}{\underset{i=1}{\sum }}{\left(RR_{i}-RR_{i-1}\right)}^{2}},$$

### 2.6 Detrending of data

Since both stress and circadian rhythms influence the activity of the sympathetic and parasympathetic nervous systems, which in turn alter heart rate oscillations, HRV, as a reflection of these oscillations, is naturally related to both stress and circadian rhythms. To better observe the contribution of stress to HRV changes, we considered removing the circadian rhythm trends from each volunteer’s HRV feature data to improve the accuracy of stress detection. The Smoothness Priors Approach (SPA) is a nonlinear detrending technique for signals (Karjalainen, 1997). This method assumes that the original data signal, i.e., the time series X , is composed of two parts:

$$X=X_{s}+X_{t},$$

where $X_{s}$ is the stationary component and $X_{t}$ is the nonlinear periodic trend component, which can be expressed as:

$$X_{t}=H\theta +\nu ,$$

where $H\in R^{N\times M}$ is the observation matrix, $\theta \in R^{M}$ is the regression parameter, ν is the observation error. We estimated the parameter θ such that ${\hat{X}}_{t}=H\hat{\theta }$ to estimate the trend component in the original data. The estimating of θ was commonly done using the method of least squares. The SPA introduced a differential term $\left\|D_{d}\left(H\theta \right)\right\|$ in the process of finding the optimal solution, minimizing it to ensure that θ filtered out the trend component of the data.

$$\theta_{\lambda }=\underset{\theta }{\mathrm{arg min}}\left\{\|H\theta -X{\|}^{2}+\lambda^{2}\|D_{d}\left(H\theta \right){\|}^{2}\right\},$$ (2)

λ represents the regularization parameter, and $D_{d}$ is the matrix of the discretized $d^{th}$ order differential operator. If the sequence X had N extreme points, represented as a column vector $R={\left[R_{1},R_{2},\ldots ,R_{N}\right]}^{⊤}\in R^{N}$ , then the first-order trend of R was given by $R_{1}=\left[R_{2}-R_{1},R_{3}-R_{2},\ldots ,R_{N}-R_{N-1}\right]\in R^{N-1}$ , and the second-order trend of R could be expressed as $R_{2}=[R_{3}-R_{2}-\left(R_{2}-R_{1}\right),R_{4}-R_{3}-\left(R_{3}-R_{2}\right),\ldots ,R_{N}-R_{N-1}-$ $\left(R_{N-1}-R_{N-2}\right)]\in R^{N-2}$ , by analogy, we could obtain the discrete representation of the trend component R of any order. Then, $D_{d}$ was calculated as follows:

$$D_{d}=\left[\begin{array}{cccc} \frac{d{\left(R_{d}\right)}_{1}}{dR_{1}} & \frac{d{\left(R_{d}\right)}_{1}}{dR_{2}} & \cdots & \frac{d{\left(R_{d}\right)}_{1}}{dR_{N}}\\ \frac{d{\left(R_{d}\right)}_{2}}{dR_{1}} & \frac{d{\left(R_{d}\right)}_{2}}{dR_{2}} & \cdots & \frac{d{\left(R_{d}\right)}_{2}}{dR_{N}}\\ \vdots & \vdots & \ddots & \vdots \\ \frac{d{\left(R_{d}\right)}_{N-d}}{dR_{1}} & \frac{d{\left(R_{d}\right)}_{N-d}}{dR_{2}} & \cdots & \frac{d{\left(R_{d}\right)}_{N-d}}{dR_{N}} \end{array}\right].$$

Furthermore, we could obtain the solution to Equation 2 as follows:

$${\hat{\theta }}_{\lambda }={\left(H^{⊤}H+\lambda^{2}H^{⊤}D_{d}^{⊤}D_{d}H\right)}^{-1}H^{⊤}X,$$

$${\hat{X}}_{t}=H{\hat{\theta }}_{\lambda },$$

${\hat{X}}_{t}$ is the estimated value of the trend component. For simplicity, let H be the identity matrix I and the order of $D_{d}$ be 2. Ultimately, we could obtain the part of the original sequence without the trend component:

$${\hat{X}}_{s}=X-H{\hat{\theta }}_{\lambda }=\left[I-{\left(I+\lambda^{2}D_{2}^{⊤}D_{2}\right)}^{-1}\right]X=LX.$$

The role of L is akin to a high-pass filter, which filters out the low-frequency components of the sequence. The parameter λ determines the frequency of the filtered signal, i.e., the frequency response. To adequately filter out the rhythmic fluctuations in HRV features, we chose the parameter λ=10000 , corresponding to a cutoff frequency of 0.0012, which was approximately a 24-h period.

### 2.7 State classification

Using the detrended HRV feature data obtained during the SCWT task as the training set, the next step involves establishing a classifier to assess stress. Extreme Gradient Boosting (XGBoost) is a classification model known for its superior accuracy compared to traditional classifiers and its ability to handle diverse types of data (Chen and Guestrin, 2016). In this study, we utilized XGBoost as a classifier to estimate stress states based on the extracted HRV features. The training process for the XGBoost model is outlined as follows:

First, we input the training dataset $T=\left\{\left(x_{1},y_{1}\right),\left(x_{2},y_{2}\right),\ldots ,\left(x_{n},y_{n}\right)\right\}$ , $x_{i}=\left(x_{i1},x_{i2},\ldots ,x_{im}\right)\in X$ , $y_{i}\in Y$ into XGBoost. $x_{ij}$ represents the $j^{th}$ feature of the $i^{th}$ sample, and $y_{i}$ represents the label of the $i^{th}$ sample, i=1,2,…,n . Then, we can build the classification model.

$${\hat{y}}_{i}=\overset{K}{\underset{k=1}{\sum }}f_{k}\left(x_{i}\right),f_{k}\in F$$

$$F=\left\{f\left(x\right)=\omega_{q}\left(x\right)\right\}\left(q:R^{k}\to T,\omega \in R^{t}\right).$$

where K represents the number of trees, and f is a function from the function space F . We use the squared error as the loss function, specifically expressed as

$$Obj=\overset{n}{\underset{i=1}{\sum }}l\left(y_{i},{\hat{y}}_{i}\right)+\overset{K}{\underset{k=1}{\sum }}\Omega \left(f_{k}\right),$$

$$\Omega \left(f_{k}\right)=\gamma T+\frac{1}{2}\lambda \overset{T}{\underset{j=1}{\sum }}\omega_{j}^{2},$$

where l is the loss function, ω is the penalty term, T is the number of leaf nodes, and γ and λ are regularization parameters used to control the number of leaf nodes and the output scores of the leaf nodes, respectively. Since the prediction results of the trees generated in each iteration during the training process are fitted to the residuals of the prediction results from the previous iteration, the model at iteration t can be expressed as

$${\hat{y}}_{i}^{\left(t\right)}={\hat{y}}_{i}^{\left(t-1\right)}+f_{t}\left(x_{i}\right).$$

Besides, the corresponding objective function can be expressed as

$$\begin{array}{l} Obj^{\left(t\right)}=\overset{n}{\underset{i=1}{\sum }}l\left(y_{i},{\hat{y}}_{i}^{\left(t\right)}\right)+\overset{t}{\underset{k=1}{\sum }}\Omega \left(f_{k}\right)\;\\ =\overset{n}{\underset{i=1}{\sum }}l\left(y_{i},{\hat{y}}_{i}^{\left(t-1\right)}+f_{t}\left(x_{i}\right)\right)+\overset{t-1}{\underset{k=1}{\sum }}\Omega \left(f_{k}\right)+\Omega \left(f_{t}\right), \end{array}$$

if we let $g_{i}=\partial_{{\hat{y}}_{i}^{\left(t-1\right)}}l\left(y_{i},{\hat{y}}_{i}^{\left(t-1\right)}\right)$ , $h_{i}=\partial_{{\hat{y}}_{i}^{\left(t-1\right)}}l\left(y_{i},{\hat{y}}_{i}^{\left(t-1\right)}\right)$ , then the objective function can be approximated as

$$Obj^{\left(t\right)}=\overset{n}{\underset{i=1}{\sum }}\left(l\left(y_{i},{\hat{y}}_{i}^{\left(t-1\right)}\right)+g_{i}f_{t}\left(x_{i}\right)+\frac{1}{2}h_{i}f_{t}^{2}\left(x_{i}\right)\right)+\overset{t-1}{\underset{k=1}{\sum }}\Omega \left(f_{k}\right)+\Omega \left(f_{t}\right).$$

Since the training for the first t−1 iterations has been completed, $l\left(y_{i},{\hat{y}}_{i}^{\left(t-1\right)}\right)$ and $\sum_{k=1}^{t-1}\Omega \left(f_{k}\right)$ are constants, and the objective function can be further simplified as

$$\begin{array}{c} Obj^{\left(t\right)}=\overset{n}{\underset{i=1}{\sum }}\left(g_{i}f_{t}\left(x_{i}\right)+\frac{1}{2}h_{i}f_{t}^{2}\left(x_{i}\right)\right)+\Omega \left(f_{t}\right)\\ \;=\overset{n}{\underset{i=1}{\sum }}\left(g_{i}f_{t}\left(x_{i}\right)+\frac{1}{2}h_{i}f_{t}^{2}\left(x_{i}\right)\right)+\gamma T+\frac{1}{2}\lambda \overset{T}{\underset{j=1}{\sum }}\omega_{j}^{2}\\ \;=\overset{T}{\underset{j=1}{\sum }}\left(\left(\underset{i\in I_{j}}{\sum }g_{i}\right)\omega_{j}+\frac{1}{2}\left(\underset{i\in I_{j}}{\sum }h_{i}+\lambda \right)\omega_{j}^{2}\right)+\gamma T, \end{array}$$

where $\omega_{j}$ represents the value of the $j^{th}$ leaf node. Let $G_{j}=\sum_{i\in I_{j}}g_{i}$ , $H_{j}=\sum_{i\in I_{j}}h_{i}$ , then $G_{j}$ and $H_{j}$ represent the sum of first derivative and second derivative of the loss function of all samples falling on the $j^{th}$ leaf node, respectively. Then the objective function can be further simplified as

$$Obj^{\left(t\right)}=\overset{T}{\underset{j=1}{\sum }}\left(G_{j}\omega_{j}+\frac{1}{2}\left(H_{j}+\lambda \right)\omega_{j}^{2}\right)+\gamma T.$$ (3)

Therefore, the optimal value of $\omega_{j}$ is $\omega_{j}=-\frac{G_{j}}{H_{j}+\lambda }$ , and substituting in Equation 3 gives

$$Obj^{\left(t\right)}=-\frac{1}{2}\overset{T}{\underset{j=1}{\sum }}\frac{G_{j}^{2}}{H_{j}+\lambda }+\gamma T.$$

In general, when building a regression tree, the best partition point of the tree is selected based on the following gain:

$$\begin{array}{cc} Gain & =Obj_{L+R}-\left(Obj_{L}+Obj_{R}\right)\\ & =\left(-\frac{1}{2}\frac{{\left(G_{L}+G_{R}\right)}^{2}}{H_{L}+H_{R}+\lambda }+\gamma T\right)\\ & \;-\left(-\frac{1}{2}\left(\frac{G_{L}^{2}}{H_{L}+\lambda }+\frac{G_{R}^{2}}{H_{R}+\lambda }\right)+\gamma \left(T+1\right)\right)\\ & =\frac{1}{2}\left(\frac{G_{L}^{2}}{H_{L}+\lambda }+\frac{G_{R}^{2}}{H_{R}+\lambda }-\frac{{\left(G_{L}+G_{R}\right)}^{2}}{H_{L}+H_{R}+\lambda }\right)-\gamma . \end{array}$$ (4)

## 3 Results

### 3.1 HRV feature analysis

**FIGURE 6:**
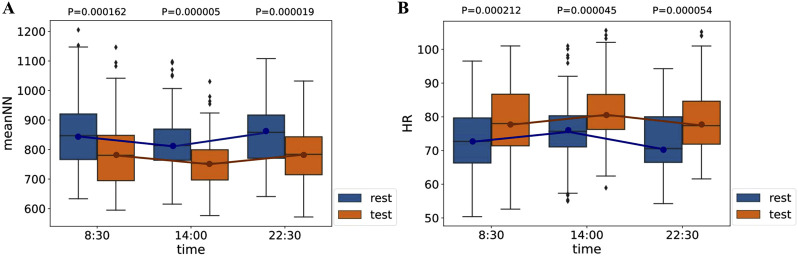
Distribution of meanNN and HR during the SCWT tasks at three time points each day: **(A)** meanNN , **(B)** HR . The blue box plots represent the data distribution for the resting state, with blue dots indicating the median. The orange box plots represent the data distribution for the stress state, with orange stars indicating the median.

Based on the RR interval data collected from 50 volunteers, we calculated the values of 10 HRV features for each participant during both the rest and stress phases across three SCWT task stages each day. Our findings indicated that the distribution of all features is influenced not only by stress but also by circadian rhythms. For instance, Figure 6 illustrates the distribution of meanNN and HR for the 50 volunteers during the rest and stress phases at different times of the day. Both meanNN and HR exhibited variations in their median values between stress and rest states over time, with similar trends in these changes. Furthermore, significant differences were observed in the distributions of meanNN and HR between the rest and stress states at the same time periods (p<0.05) . Notably, around 8:30 and 22:30, the distributions of meanNN and HR during the stress state overlapped with those during the rest state observed at approximately 14:00.

This finding indicates that meanNN and HR are sensitive indicators of stress. Additionally, when assessing stress based on these HRV features, it is essential to further mitigate the influence of circadian rhythms. Moreover, the effect of circadian rhythms on the data distribution was also evident for the remaining eight features (Supplementary Figure S1).

### 3.2 Circadian rhythm analysis

**FIGURE 7:**
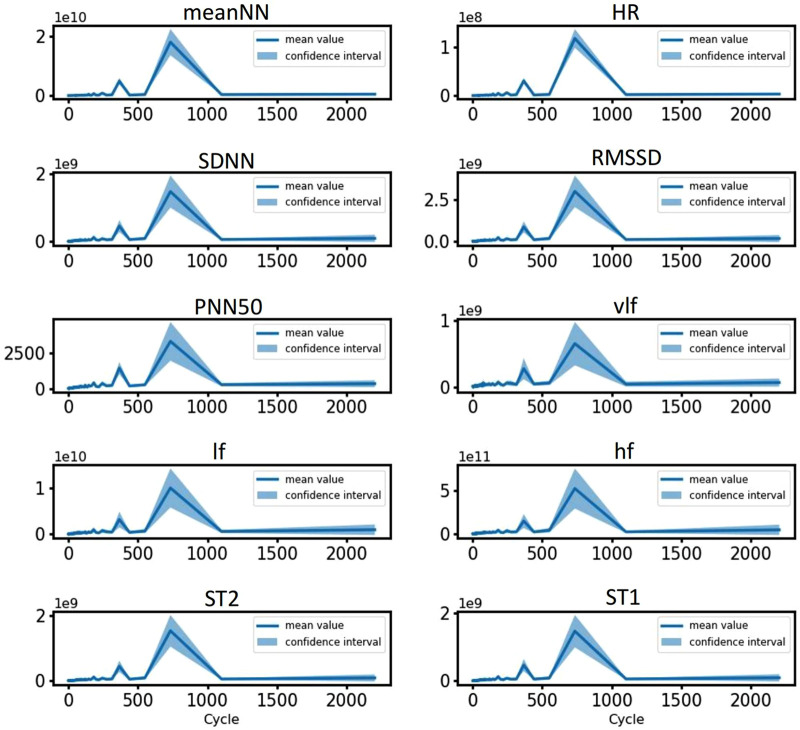
Period spectrum plots of HRV features for all volunteers. The blue line represents the average amplitude, and the shaded area corresponds to the interval estimate.

Given that circadian rhythms influence all HRV features, we conducted a detailed analysis of the specific periods associated with each feature, verifying the presence of circadian rhythms through sinusoidal fitting. Using the 3-day RR interval data from all volunteers, we extracted the values of 10 HRV features every 2 min. Subsequently, we applied FFT to determine the periods and amplitudes of all features (Cooley and Tukey, 1965). We then calculated the mean values of the 10 features for each period across all volunteers, allowing us to perform interval estimation of the overall mean. The amplitude of each feature exhibited significant peaks around 360 min and 720 min, with the 95% interval estimates aligning closely with the trends in the mean values (as shown in Figure 7). These results indicate that each feature demonstrated periodicity, consistently present within the population. Since feature values were extracted at 2-min intervals, the actual corresponding periods corresponded to 12 h and 24 h. This suggests substantial 24-h fluctuations in each feature, alongside potential 12-h fluctuations during daytime hours.

**FIGURE 8:**
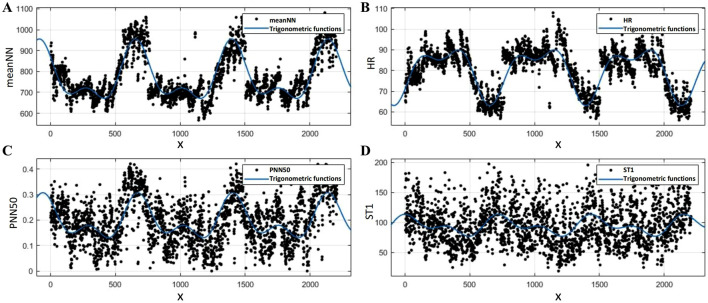
Results of the second-order sine function fitting for HRV features: **(A)** meanNN , **(B)** HR , **(C)** PNN50 , **(D)** ST1 .

Furthermore, to quantify the differences in circadian rhythms both between individuals and within the same individual across different days, we employed a second-order sine function to fit the time series of each feature for all volunteers collectively.

$$y=A_{0}+A_{1}\sin\left(\omega x\right)+B_{1}\cos\left(\omega x\right)+A_{2}\sin\left(2\omega x\right)+B_{2}\cos\left(2\omega x\right),$$ (5)

where $A_{0}$ is a constant, $A_{1}$ , $B_{1}$ , $A_{2}$ and $B_{2}$ are the amplitudes, and ω is the frequency. These parameters described the diurnal dynamic variations of HRV features, which were fitted using MATLAB according to Equation 5 to obtain the results. As illustrated in Figure 8, the fitting results for meanNN , HR , PNN50 and ST1 correspond closely with the variation trends of the original data over time. The x-axis denotes time, with x values in the intervals 1–501, 735–1,242, and 1,472–1978 corresponding to the time range of 7:30 a.m. to 12:00 a.m., while the remaining intervals represent the hours from 12:00 a.m. to 7:30 a.m. These features exhibit significant diurnal rhythms. For instance, meanNN initially decreases, then stabilizes, and finally increases throughout the day, exhibiting an opposite trend at night. Notably, we also observed that the feature values at night were either significantly higher or significantly lower than those during the day. This results in substantial differences between the distributions of rest and stress state data collected during the day compared to those collected at night. If HRV feature data from different times are input into the same classifier, it may not accurately assess the stress state. For the remaining six features, we similarly applied a second-order sine function for fitting and also observed clear rhythmicity.

Additionally, we fitted the 10 feature data for each volunteer on each day separately, obtaining the corresponding fitted parameter values for each individual on each day ( $A_{1}$ , $B_{1}$ , $A_{2}$ and $B_{2}$ ). To compare the variations in HRV within an individual over 3 days and across all the participants, we then calculated the mean values of each parameter across all days for the 50 volunteers (mean) , as well as the mean standard deviation of each parameter for each individual over the 3 days $\left(std_{1}\right)$ , and the overall standard deviation of each parameter across all days $\left(std_{2}\right)$ (as shown in Table 1). By comparing $std_{1}$ and $std_{2}$ , we found that the differences in rhythmic parameters within the same volunteer across different days were relatively small.

**TABLE 1:** Parameter results of the second-order sinusoidal fitting for HRV feature data of all volunteers. For each fitted parameter, the first row represents the mean value of the parameters obtained by fitting 3 days of data together for each volunteer, resulting in the mean value for 50 volunteers. The second row represents the mean of the standard deviations of the fitting parameters for each volunteer over 3 days. The third row represents the standard deviation of the 50 parameters obtained by fitting 3 days of data together for each volunteer.

Parameter	Statistic	meanNN	HR	SDNN	RMSSD	PNN50
$A_{1}$	mean	77.60	−7.20	−18.72	−32.94	−0.08
	$std_{1}$	11.08	0.99	4.77	10.02	0.09
	$std_{2}$	41.98	4.09	19.67	28.69	0.22
$B_{1}$	mean	−61.04	4.68	11.99	16.53	−0.13
	$std_{1}$	10.80	1.12	3.378	13.45	0.18
	$std_{2}$	35.18	2.28	21.08	22.43	0.47
$A_{2}$	mean	2.28	−0.43	−1.21	−4.33	−0.03
	$std_{1}$	1.16	1.23	0.69	2.33	0.05
	$std_{2}$	27.12	2.04	13.74	18.56	0.12
$B_{2}$	mean	−37.29	3.40	11.96	13.34	0.06
	$std_{1}$	11.17	1.55	3.25	3.54	0.07
	$std_{2}$	22.41	2.09	17.60	24.69	0.16

Parameter	Statistic	vlf	lf	hf	ST2	ST1
$A_{1}$	mean	−9.44	−82.62	−551.8	−23.39	−18.42
	$std_{1}$	2.28	19.51	132.1	3.51	1.00
	$std_{2}$	16.13	97.15	341.34	20.39	22.85
$B_{1}$	mean	10.08	52.62	317.2	11.74	14.91
	$std_{1}$	2.35	22.01	162.9	1.08	9.21
	$std_{2}$	22.33	70.34	458.0	15.95	19.51
$A_{2}$	mean	−1.18	−20.15	−102.4	−3.09	0.95
	$std_{1}$	0.43	8.14	23.0	1.31	0.56
	$std_{2}$	10.24	55.80	151.5	13.19	14.42
$B_{2}$	mean	10.08	34.07	204.2	9.49	11.53
	$std_{1}$	2.82	13.75	63.9	9.20	2.92
	$std_{2}$	18.32	69.02	195.9	17.56	20.11

These results indicate that the 10 HRV features exhibit a distinct circadian rhythm trend, and the specific rhythm-related parameters obtained can be utilized for the subsequent removal of these trends. Moreover, the rhythmic patterns of HRV parameters exhibit minimal fluctuations within individuals over three consecutive days, reflecting relative intra-individual stability. Consequently, data from the previous day can be utilized as a reference for detrending circadian rhythm trends when assessing stress throughout the day. However, considerable inter-individual variability is observed, suggesting that the data collected from all volunteers encompass a certain degree of diversity.

### 3.3 Detrended HRV feature analysis

**FIGURE 9:**
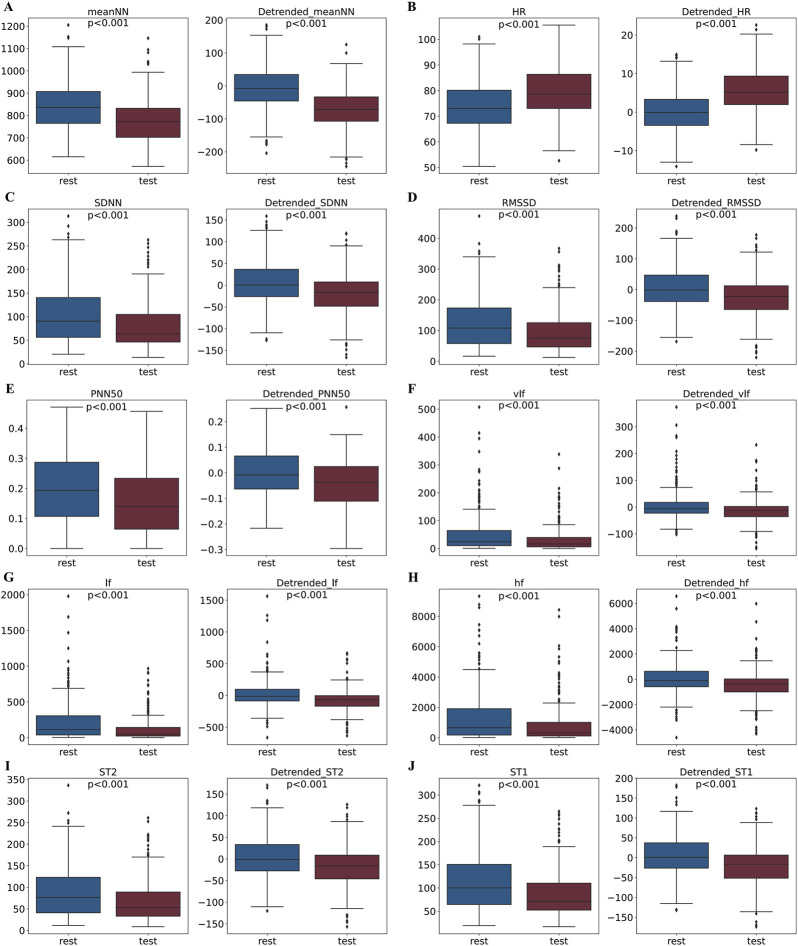
Comparison of feature distributions between rest and stress states before and after removing circadian rhythm trends. For each module, the left panel shows the feature distribution before trend removal, and the right panel shows the distribution after trend removal.

Considering the influence of circadian rhythms on HRV features in stress assessment, we further investigated the effects of removing these trends from the HRV feature data. The distribution of the 10 HRV feature values for all volunteers during the rest and stress phases of the SCWT experiment is illustrated in Figure 9. Each panel presents two graphs: the left graph displays the value distribution of each feature during the rest and stress phases before detrending, while the right graph shows the distribution after detrending. Notably, distinct differences in the distribution of each feature between the rest and stress phases are observed both before and after the detrending process (p<0.05) . To further investigate, we examine whether detrending enhances these differences, making the separation between the two states more pronounced.

First, we quantified the differences in value distributions of each feature between groups and within groups during the rest and stress phases using the following equations:

$$SSA=n_{1}{\left({\hat{x}}_{1}-\hat{x}\right)}^{2}+n_{2}{\left({\hat{x}}_{2}-\hat{x}\right)}^{2},$$

$$SSE=\overset{n_{1}}{\underset{j=1}{\sum }}{\left(x_{1j}-{\hat{x}}_{1}\right)}^{2}+\overset{n_{2}}{\underset{j=1}{\sum }}{\left(x_{2j}-{\hat{x}}_{2}\right)}^{2},$$

where $n_{1}$ and $n_{2}$ represent the sample number of the feature data during the rest and stress phases, respectively, ${\hat{x}}_{1}$ and ${\hat{x}}_{2}$ represent the means of the feature data during the rest and stress phases, respectively, and $\hat{x}$ is the overall mean of the features. Additionally, the degrees of freedom for SSA and SSE are 1 and $n_{1}+n_{2}-2$ , respectively. Then, we can calculate the F-statistic for each feature using the following formula:

$$F=\frac{SSA}{SSE/\left(n_{1}+n_{2}-2\right)}.$$

The larger the F value, the greater the difference in data distribution between the rest and stress phases for the corresponding feature. The calculated F -statistic results for each feature before and after detrending are presented in Table 2. All features exhibited significantly increased F values following detrending, indicating that the distribution differences between the rest and stress phases became more pronounced.

**TABLE 2:** The F-statistics for the distribution of each HRV feature in resting and stress states. The first column shows the results for HRV feature data without removing circadian rhythm trends, while the second column shows the results for HRV feature data after removing circadian rhythm trends.

Features	Original F-statistic	Detrended F-statistic
meanNN	54.410	151.796
HR	47.975	152.578
SDNN	23.514	33.829
RMSSD	19.352	26.978
PNN50	19.465	30.536
vlf	15.438	20.984
lf	23.298	30.256
hf	16.811	22.750
ST2	19.391	27.030
ST1	25.722	37.384

In summary, the influence of circadian rhythms leads to significant overlap in the HRV feature distributions for stress and rest states throughout the day. By removing the circadian rhythm trend, the differences in HRV features between stress and rest states are enhanced.

### 3.4 Stress assessment using XGBoost

To investigate the impact of detrended HRV feature data obtained during the SCWT task on stress assessment throughout the entire time period, we trained XGBoost models as classifiers. We determined the optimal hyperparameters of the model through a grid search approach. Specifically, we adjusted each parameter individually within a predefined range and utilized these parameters to train the model. In each training session, 20% of the training dataset was reserved as the test dataset, with the F1 score serving as the evaluation criterion for model performance. Ultimately, we identified the parameter settings that yielded the highest accuracy on the test dataset as the optimal values for each hyperparameter.

**FIGURE 10:**
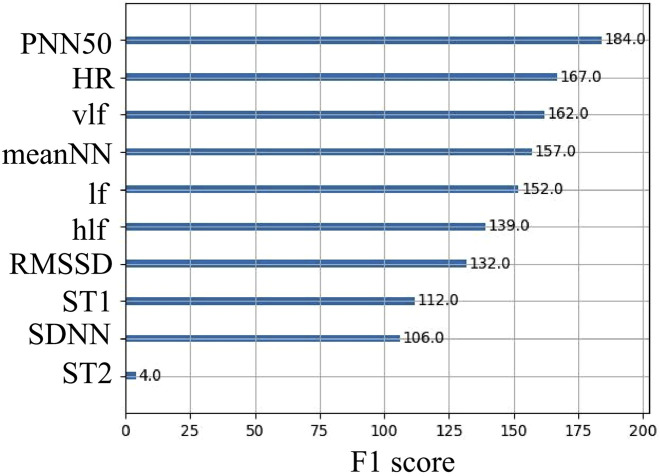
Ranking of the importance of the 10 HRV features obtained using XGBoost.

Additionally, XGBoost, as a gradient boosting tree model, has the capability to output feature importance. Specifically, for each node of each decision tree, XGBoost calculates the gain resulting from splitting the node, which reflects the extent to which the split enhances model performance (Equation 4). The importance score for each feature is then obtained by weighting and summing the results across all decision trees, followed by averaging these scores (as shown in Figure 10). To enhance the model’s accuracy, we ultimately selected the top seven important features for classification.

**FIGURE 11:**
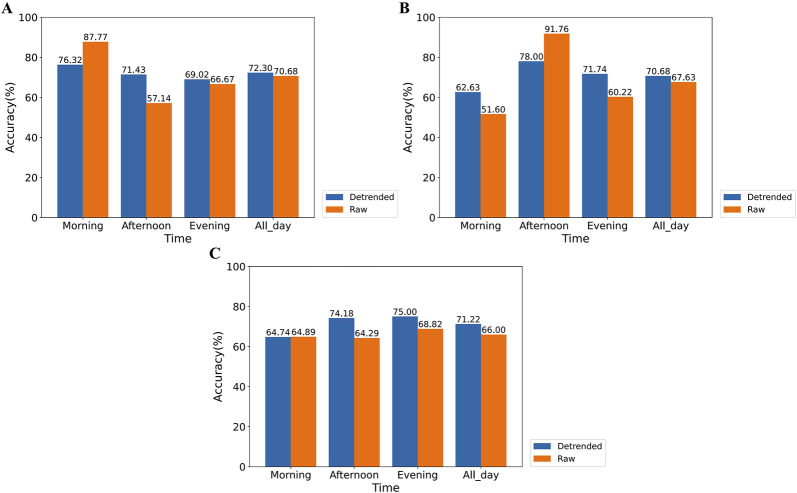
Comparison of the accuracy of stress assessment for different time periods using XGBoost classifiers trained on SCWT experimental data for each time period. This includes classifiers trained using only morning **(A)**, afternoon **(B)**, or evening **(C)** experimental data.

The classification accuracy of the XGBoost model trained on various HRV feature datasets is depicted in Figure 11. Figures 11A–C illustrate the accuracy of classifiers trained on HRV feature data obtained from the SCWT experiments conducted in the morning, afternoon, and evening, respectively, using both raw data and detrended data for stress assessment across different time periods. For the raw dataset, classifiers trained on data from a specific SCWT task exhibit high accuracy in assessing stress during that time period. However, these classifiers show significantly lower accuracy in assessing stress for other SCWT tasks compared to those trained on detrended data. Furthermore, classifiers trained on detrended data from each SCWT task demonstrate higher overall accuracy in assessing stress across all SCWT tasks compared to those trained on raw data (morning: +1.62%, afternoon: +3.05%, evening: +5.22%) and exhibit less variation in accuracy across different time periods.

These results indicate that the presence of circadian rhythms adversely affects stress detection throughout the day, while detrending significantly enhances classification accuracy.

**FIGURE 12:**
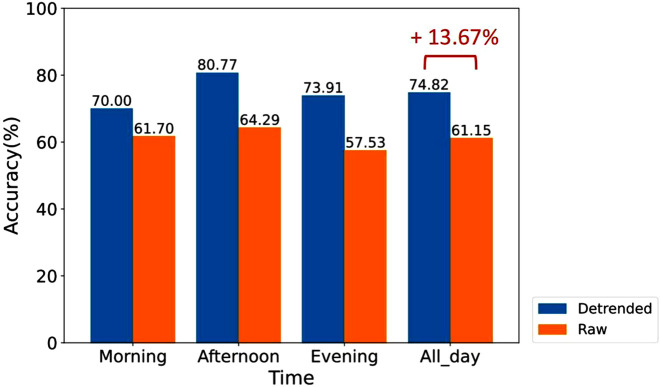
Comparison of the accuracy of stress assessment for different time periods using XGBoost classifiers trained on a dataset that randomly samples one-third of the integrated data from different SCWT tasks.

Furthermore, we trained the XGBoost model using integrated HRV feature data from all SCWT tasks (as shown in Figure 12). To ensure a consistent sample size in the training dataset, we randomly selected one-third of the data from each SCWT task and combined them to form the training dataset.

For the non-detrended data, the classifier trained on the integrated dataset from all SCWT tasks exhibited overall lower accuracy in assessing stress compared to classifiers trained on data from each individual SCWT task. This finding indicates that simply integrating data from different time periods into a single training dataset does not enhance the overall accuracy of stress assessment across all periods, highlighting the significant impact of circadian rhythms.

In contrast, for the detrended data, the classifier trained on integrated data from different SCWT tasks maintained a high classification accuracy. As illustrated in Figure 12, the accuracy of the classifier reaches 74.82% for overall stress assessment across all SCWT tasks, reflecting a notable improvement of 13.67% compared to the classifier trained on the raw data. This substantial enhancement highlights the critical role of detrending in improving stress recognition performance and underscores its necessity for achieving more reliable and accurate analyses.

Notably, the detrended data used for training the stress classification model was collected from all volunteers. In general, HRV varies among individuals, with differences potentially influenced by factors such as age. For instance, the results presented in Table 1 demonstrate the diversity of circadian rhythm trends in HRV features across individuals. To further investigate this, we examined the impact of inter-individual HRV differences on the classification accuracy of the XGBoost model trained on detrended data.

**FIGURE 13:**
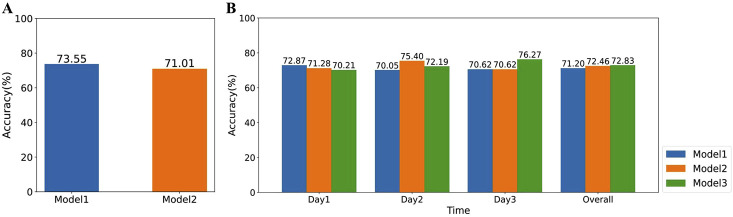
The impact of volunteers’ age and familiarity with the experiment on the accuracy of stress assessment. **(A)** Comparison of the accuracy of XGBoost classifiers trained on data from volunteers of different age groups. Model1 and Model2 represent classifiers trained on data from volunteers aged 26 and below and those aged above 26, respectively. **(B)** Comparison of the accuracy of XGBoost classifiers trained on data from different days. Model1, Model2, and Model3 represent classifiers trained on volunteer data collected on the first, second, and third days, respectively.

Since nearly half of the recruited volunteers were 26 years old or younger (as shown in Figure 2A), we further investigated the impact of HRV differences across age groups on the classification accuracy. We categorized all participants into two groups: Group 1 (age ≤ 26) and Group 2 (age > 26). In Figure 13A, “Model1” and “Model2” represent the overall classification accuracy of stress assessment across all time periods for all participants, obtained from classifiers trained on detrended data from volunteers in Group 1 and Group 2, respectively. Due to the relatively concentrated age distribution of our recruited volunteers and the removal of circadian rhythm trends, which partially reduces HRV feature differences across age groups, the overall accuracy of stress assessment using classifiers trained on detrended data from these two groups remains comparable. However, it is important to note that when there is a larger age disparity among participants, the impact of age-related HRV differences on stress assessment accuracy should be carefully considered.

Moreover, during the data collection phase, as volunteers become more familiar with the experimental procedure, their stress levels and patterns may change. To investigate the impact of this familiarity on the accuracy of the final classifier, we conducted further analyses. In general, volunteers’ familiarity with the experiment gradually increases from the first to the third day. To examine this effect, we trained three classifiers separately using detrended SCWT task data from the first, second, and third days, respectively. We then evaluated the accuracy of these classifiers in assessing stress levels for all volunteers on each individual day, as well as their overall accuracy across the 3 days. As shown in Figure 13B, classifiers trained on detrended data from a specific day achieved consistently high accuracy in stress assessment for other days, and the overall accuracy of the three classifiers remained relatively similar. These findings suggest that changes in stress levels and patterns due to increased familiarity with the experiment have a minimal impact on the accuracy of the final classifier in our study.

## 4 Discussion

This study analyzed the rhythmicity of HRV features influenced by the autonomic nervous system and examined its impact on stress detection throughout the day. First, outlier removal was conducted on the RR interval data collected from 50 volunteers over three consecutive days using a confidence ellipse with a sliding window. Next, 10 HRV features were extracted. The rhythmic periods of these HRV features were calculated using the FFT algorithm, and the circadian rhythm trends were removed using the SPA method. Finally, XGBoost classifiers were trained on the detrended HRV feature data from various SCWT tasks to assess psychological states.

Specifically, during the preprocessing step, a confidence ellipse with a sliding window was applied to the collected RR interval data to effectively eliminate outlier data points. The exclusion of these outliers reduced the average CV of the RR interval data from 0.28 to 0.20, thereby enhancing data quality and reliability. Then, in the analysis of circadian rhythms in HRV features, we employed FFT to compute the periods and amplitudes of HRV features, revealing distinct 12-h and 24-h periodic components. Additionally, a second-order sine function was utilized to model the diurnal fluctuations of these features. Subsequently, to eliminate circadian rhythmic trends in HRV features, we applied the SPA method to filter out these trends in order to more clearly observe the contribution of stress to HRV variations. After detrending, the distribution differences of each HRV feature between resting and testing states were more distinctly evident. Finally, XGBoost classifiers were trained using the data collected from the SCWT task to evaluate stress. The classifier’s accuracy improved by 13.67% when trained on the detrended data.

When analyzing the circadian rhythmic trends in HRV features, we found that the day-to-day variability within the same individual was smaller than the variability between individuals. This suggests that circadian rhythm in individual experimental data can be removed by using data from the previous day as a reference. However, these HRV indicators are generally influenced by a variety of internal and external factors. In this study, the participants were primarily young students and teachers with relatively regular daily routines. Given the sample size of 50, further stratification based on factors such as menstrual cycle and menopausal status in females, individual intelligence levels, personality traits, occupations, and day-night work patterns would result in subgroups too small to yield reliable conclusions. Nevertheless, the potential impact of these factors should not be overlooked. Studies have found that children and older adults exhibit longer reaction times and higher error rates when performing the Stroop task (Ménétré and Laganaro, 2023). In a small-scale study, it was found that men were consistently slower than women across trial blocks by approximately 46 milliseconds, although their error rates did not differ significantly (Mekarski et al., 1996). In cognitive function tests, patients with Alzheimer’s disease (AD) performed significantly worse than the normal control group on the Mini-Mental State Examination (MMSE) scores and various sub-scores of the Stroop test. Additionally, certain indices showed a significant correlation with glucose metabolism in specific regions of the prefrontal cortex (Yun et al., 2011). Studies on sleep deprivation have found that after a night of sleep loss, participants exhibit significantly prolonged reaction times in the Stroop task (Cain et al., 2011). The potential roles of these factors in stress detection based on daily HRV features will be an important focus of future research.

Additionally, due to the combined influence of stress and circadian rhythms on HRV, we ultimately used the HRV data with the circadian rhythmic trends removed to train the classifier for stress assessment. It is important to note that studies have shown stress can also affect circadian rhythms (Weibel et al., 2002; Tahara et al., 2016), and this influence should be considered when removing circadian rhythm components from HRV. However, in our data collection, the stress induced by the SCWT test was short in duration and the stressors were controlled, minimizing the impact of stress on circadian rhythms (Thompson et al., 2013).

In the comparison between the XGBoost classifiers trained on detrended data and non-detrended data, when SCWT experimental data from only the morning, afternoon, or evening were used as the training dataset, classifiers trained on non-detrended data from each SCWT task demonstrated high accuracy, but only for stress assessment within the corresponding time period. This finding suggests that previous studies, which collected data during fixed time periods, may have partially reduced the influence of circadian rhythms (Hemakom et al., 2024; da Estrela et al., 2020). However, stress classification models developed in this way are less reliable for detecting stress at other times of the day. In contrast, classifiers trained on detrended HRV data from each SCWT task exhibited consistently high accuracy in stress classification across all SCWT tasks and achieved higher overall accuracy in stress assessment throughout the day (morning: +1.62%, afternoon: +3.05%, evening: +5.22%). These results indicate that classifiers trained on detrended HRV feature data provide a more robust and effective assessment of stress across different time periods.

Furthermore, when the data from different time periods of the SCWT task were integrated as the training set, classifiers trained on the non-detrended data exhibited low accuracy in stress assessment for each SCWT task, confirming the negative impact of circadian rhythms on stress evaluation. In contrast, classifiers trained on the detrended data demonstrated significantly higher accuracy in stress assessment across all SCWT tasks compared to those trained on non-detrended data, achieving an overall accuracy improvement of 13.67%. The significant improvement in accuracy suggests that fluctuations in HRV features caused by intrinsic circadian rhythms may mask the changes induced by short-term stress, particularly when the stress levels are not extreme. Directly analyzing non-detrended data may overlook many stress-related probnlems, potentially leading to severe adverse consequences. Additionally, the ability of the combined detrended data from different time periods to produce highly accurate classifiers allows for more flexible data collection times, enhancing the convenience of the experimental process. Moreover, existing studies have also evaluated stress using physiological indicators beyond HRV, such as electroencephalography and functional near-infrared spectroscopy (Al-Shargie et al., 2017; 2016). Since most physiological indicators are influenced by circadian rhythms (Stangherlin et al., 2020), our approach can be widely applied to these stress assessment methods, thereby improving both the accuracy of stress detection and the convenience of data collection.

Although our findings demonstrate that removing circadian rhythms from HRV data can enhance stress detection accuracy across different time periods, there are still some limitations. Due to constraints in experimental conditions and the available dataset, this study did not directly perform stress recognition across all time periods. Furthermore, the final classifier we obtained can only identify the presence or absence of stress, without providing a specific stress level. We aim to address this gap in future research. In the SCWT task, the need to repeat the experiment following a failure may be influenced by the participant’s personality traits, which could affect their experienced stress levels. Additionally, repeated exposure to the experimental procedure may introduce a training effect. These limitations will be thoroughly addressed in future large-scale studies. Moreover, we treated pulse rate variability (PRV) derived from smartwatch data as equivalent to HRV. However, prior studies have shown that PRV and HRV may differ under certain conditions (Mejía-Mejía et al., 2020). To further improve the accuracy of stress detection, these differences should be carefully considered.

## 5 Conclusion

In this study, HRV features with circadian rhythm trends removed were used to assess stress throughout the day. We found that the extracted HRV features exhibited periodic fluctuations with 12-h and 24-h cycles, causing overlaps between stress-induced HRV feature distributions during one time period and resting states during another, which reduced classification accuracy. Additionally, circadian rhythm variations between different days for individuals were considered negligible. Compared to non-detrended HRV data, XGBoost classifiers trained on detrended data from each SCWT task demonstrated improved overall accuracy in stress assessment across all SCWT tasks (morning: +1.62%, afternoon: +3.05%, evening: +5.22%), with smaller differences in accuracy across different tasks. Moreover, when data from different time periods were combined to train the classifier, the overall accuracy of the classifier trained on non-detrended data for all SCWT tasks significantly decreased. In contrast, the classifier trained on detrended data maintained high overall accuracy, outperforming the non-detrended classifier by 13.67%. These findings indicate that using detrended HRV feature data is an effective method for assessing stress throughout the day and allows for more flexible data collection.

## Data Availability

The raw data supporting the conclusions of this article will be made available by the authors, without undue reservation.
