# Supplementary material for: Heart rate variability with circadian rhythm removed achieved high accuracy for stress assessment across all times throughout the day
Source: Front Physiol. 2025 Apr 14;16:1535331. doi: 10.3389/fphys.2025.1535331 (PMC12034550; doi:10.3389/fphys.2025.1535331)
Supplement: Supplementary file 1 [file DataSheet1.pdf]

# Supplementary Material

This PDF file presents detailed information on power spectral density estimation, along with distribution charts of eight HRV features during rest and stress states over different time intervals.

## 1 POWER SPECTRUM ESTIMATION

In this work, we used Auto Regression Model (AR) to analyze the frequency domain characteristics of HRV. The expression of the AR model is

$$R(n) = -\sum_{k=1}^{p} a_k RR(n-k) + u(n), \tag{S1}$$

where $R(n)$ is the predicted sequence, $RR(n-k)$ is the input sequence, $p$ is the order of the AR model, $u(n)$ is the white noise sequence, and $a_k$ $(k = 1, 2, \ldots, p)$ are the parameters of the AR model. Then, through the z-transform, we can obtain the system function of the model.

$$H(z) = \frac{1}{A(z)} = \frac{1}{1 + \sum_{k=1}^{p} a_k z^{-k}}. \tag{S2}$$

Furthermore, the estimated value of the power spectral density can be obtained.

$$P_{xx}(\omega) = \sigma_\omega^2 |H(e^{j\omega})|^2 = \frac{\sigma_\omega^2}{|1 + \sum_{k=1}^{p} a_k e^{jk\omega}|^2}, \tag{S3}$$

where $H(e^{j\omega})$ is the frequency response function, and $a_k$ and $\sigma_\omega$ are the parameters to be determined in the AR model. In this study, we use the Burg method to estimate $a_k$ and $\sigma_\omega$.

First, the initial conditions can be determined as

$$e_0(n) = b_0(n) = R(n), 0 \le n \le N-1, \tag{S4}$$

$$\sigma_0^2 = \frac{1}{N} \sum_{n=0}^{N-1} RR^2(n). \tag{S5}$$

Then, we calculate the reflection coefficients as

$$K_k = a_{kk} = -\frac{2\sum_{n=p}^{N-1} [e_{p-1}(n) b_{p-1}(n-1)]}{\sum_{n=p}^{N-1} [e_{p-1}^2(n) + b_{p-1}^2(n-1)]}. \tag{S6}$$

Next, the values of $a_{ki}$, $e_k(n)$, $b_k(n)$, and $\sigma_k^2$ can be calculated as

$$a_{ki} = a_{k-1,i} + a_{kk} a_{k-1,k-i}, \tag{S7}$$

$$e_k(n) = e_{p-1}(n) + K_p b_{p-1}(n-1), \tag{S8}$$

$$b_k(n) = b_{p-1}(n) + K_p e_{p-1}(n), \tag{S9}$$

$$\sigma_k^2 = (1 - K_p^2)\sigma_{k-1}^2. \tag{S10}$$

Let $k = k + 1$ and repeat the above calculation steps until $k = p$. Finally, the HRV power spectral density $PSD(\omega)$ can be obtained, allowing for the extraction of HRV features.

## 2 ANALYSIS OF THE REMAINING HRV FEATURES

Figure S1 shows the distribution of the remaining 8 HRV features for the 50 volunteers during rest and stress phases at different time periods. The median values of the distribution of all these features during the stress state and the rest state changed over time. Besides, at the same time period, there were significant differences in the distribution of these features between the rest and stress states ($p < 0.05$). However, around 8:30 and 22:30, the distribution of these features during the rest state overlapped with the distribution during the stress state around 14:00. These results indicate that the influence of circadian rhythms on these HRV features is significant and cannot be ignored.

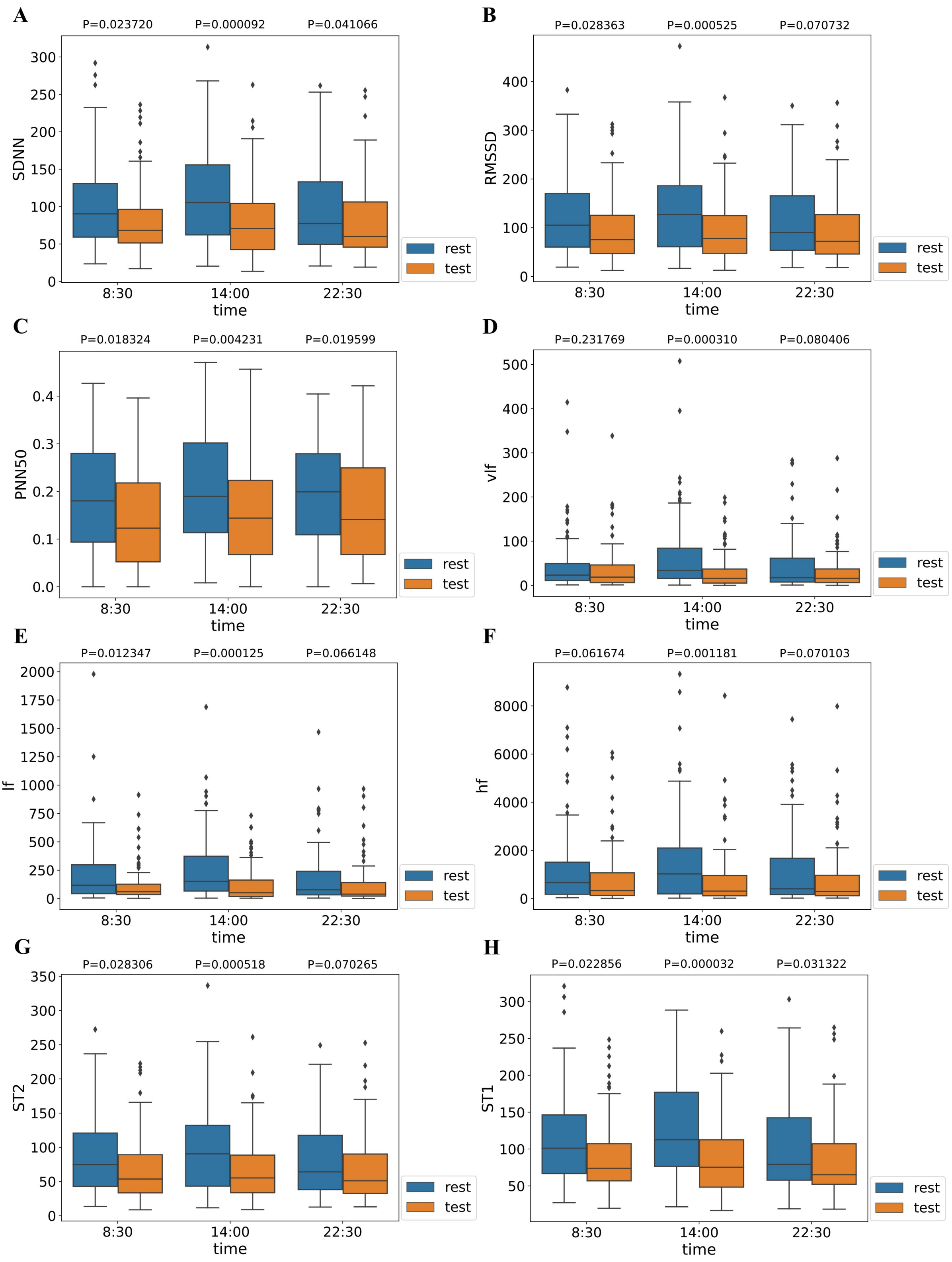


**Figure S1.** Distribution of HRV features during the SCWT tasks at three time points each day: (A) $SDNN$, (B) $RMSSD$, (C) $PNN50$, (D) $vlf$, (E) $lf$, (F) $hf$, (G) $ST2$, (H) $ST1$. The blue box plots represent the data distribution for the resting state, and the orange box plots represent the data distribution for the stress state.
